# Supplementary material for: Clinical and pathological implications of the presence of MECA-79-expressing tumor cells in pathological stage IA lung adenocarcinoma
Source: PLoS One. 2025 Oct 16;20(10):e0323233. doi: 10.1371/journal.pone.0323233 (PMC12530585; doi:10.1371/journal.pone.0323233)
Supplement: S1 Table — (DOCX) [file pone.0323233.s001.docx]

**Supplementary Materials**

**Clinical and pathological implications of the presence of MECA-79-expressing tumor cells in pathological stage IA lung adenocarcinoma**

**S1 Table. Multivariate Cox proportional hazards models including two* candidate factors for recurrence-free survival.**

In exploratory analyses, the presence of MECA-79^+^ tumor cells showed a tendency toward association with worse recurrence-free survival in some multivariable Cox proportional hazards regression models; however, this association was not consistent and disappeared in the model incorporating vascular invasion.

|  | *Multivariate model 1* | | | *Multivariate model 2* | | | *Multivariate model 3* | | |
| --- | --- | --- | --- | --- | --- | --- | --- | --- | --- |
| *Characteristics* | **HR** | **95% CI** | ***P*-value** | **HR** | **95% CI** | ***P*-value** | **HR** | **95% CI** | ***P*-value** |
| Body mass index | 0.88 | 0.78–0.99 | 0.026 |  |  |  |  |  |  |
| IASLC grade of LUAD:  3 vs. 1–2 |  |  |  | 2.11 | 0.99–4.48 | 0.052 |  |  |  |
| Vascular invasion: (+) vs. (−) |  |  |  |  |  |  | 2.93 | 1.31–6.57 | 0.009 |
| STAS: (+) vs. (−) |  |  |  |  |  |  |  |  |  |
| MECA-79^+^ tumor cells: (+) vs. (−) | 3.20 | 1.06–9.67 | 0.040 | 4.00 | 1.37–11.7 | 0.011 | 2.79 | 0.93–8.35 | 0.067 |

|  | *Multivariate model 4* | | |
| --- | --- | --- | --- |
| *Characteristics* | **HR** | **95% CI** | ***P*-value** |
| Body mass index |  |  |  |
| IASLC grade of LUAD:  3 vs. 1–2 |  |  |  |
| Vascular invasion: (+) vs. (−) |  |  |  |
| STAS: (+) vs. (−) | 4.24 | 2.55–23.1 | < 0.001 |
| MECA-79^+^ tumor cells: (+) vs. (−) | 4.07 | 1.40–11.9 | 0.010 |

*Given the number of recurrence or death events was 30 as described in the cohort (as described in the main text), two independent variables, including the presence of MECA-79^+^ tumor cells, were selected for multivariable Cox model to minimize overfitting and adhere to the “rule of ten” events per variable [1] Abbreviations: HR, hazard ratio; CI, confidence interval; IASLC, International Association for the Study of Lung Cancer; LUAD, lung adenocarcinoma; STAS, spread through air spaces.

Reference:

Concato J, Peduzzi P, Holford TR, Feinstein AR (1995) Importance of events per independent variable in proportional hazards analysis. I. Background, goals, and general strategy. J Clin Epidemiol 48: 1495–1501.
