# Supplementary material for: Clinical and pathological implications of the presence of MECA-79-expressing tumor cells in pathological stage IA lung adenocarcinoma
Source: PLoS One. 2025 Oct 16;20(10):e0323233. doi: 10.1371/journal.pone.0323233 (PMC12530585; doi:10.1371/journal.pone.0323233)
Supplement: Attachment — Submitted filename: report_10.1101+2025.04.08.25325444.pdf [file pone.0323233.s004.pdf]

ScreenIT Report

This is an automated report for 10.1101+2025.04.08.25325444.pdf. This report was solicited by the PLOS One editorial team and provided by ScreenIT.

ScreenIT is an independent group of scientists developing automated tools that analyze academic papers. A set of automated tools screened your submitted manuscript and provided the report below. Each tool was created by your academic colleagues with the goal of helping authors.

The tools look for factors that are important for transparency, rigor and reproducibility, and we hope that the report might help you to improve reporting in your manuscript. Within the report you will find links to more information about the items that the tools check. These links include helpful papers, websites, or videos that explain why the item is important.

While our screening tools aim to improve and maintain quality standards they may, on occasion, miss nuances specific to your study type or flag something incorrectly. Each tool has limitations that are described on the ScreenIT website. The tools screen the main file for the paper; they are not able to screen supplements stored in separate files.

Please note that the Academic Editor had access to these comments while making a decision on your manuscript. The Academic Editor may ask that issues flagged in this report be addressed.

If you would like to provide feedback on the ScreenIT tool, please email the team at ScreenIt@bih-charite.de. If you have questions or concerns about the review process, please contact the PLOS One office at plosone@plos.org.

How to read the report:

- no action required
- review for potential issues
- a critical issue was found by a tool
- may not be applicable to your paper

- more information about this item can be obtained using this link

Rigor

	Ethics	IRB: "*This study was approved by the Kansai Medical University Hospital Research Ethics Committee (approval number: 2016662; approval date: September 12, 2016).*"<br>Consent: "*The requirement for informed consent was waived as this retrospective study analyzed anonymized data and posed minimal risk to participants.*"
	Inclusion and Exclusion Criteria	"*The inclusion criteria were a pathological diagnosis of primary invasive LUAD, pathological stage IA (as per the eighth edition of the TNM classification system) [12], complete resection (R0) with lobectomy or greater, and tissue available for this study.*"
	Flow charts and attrition	Thank you for including a study flow chart and information about attrition and exclusions to help readers evaluate the risk of bias.<br><br>Sentence about attrition: "*Based on these criteria, 195 patients who had undergone pulmonary lobectomy with lobe-specific lymph node dissection were included for analysis in this study.*"
	Sex as a biological variable	Not detected. Please consider reporting the sex of human participants, animals or cells, if you haven't done so.
	Subject Demographics	Age: not detected.<br>Weight: not detected.
	Randomization	Not detected. If you performed an experimental study, please specify whether participants, animals or samples were randomly assigned to treatments or groups, and specify the randomization procedure.
	Blinding	Not detected. Please specify whether blinding was used at various phases of the experiment (e.g., blinding of patients, caregivers, outcome assessments, data analysis).
	Power Analysis	Not detected. Please state how you determined the sample size for the study. If you

?		performed a power or sample size calculation, please provide details.
n/a	Replication	not required.

Generated by *Barzooka (flow charts)*, *SciScore (everything else)*.
SciScore only screens the text of the methods section; we apologize if the tool has missed items reported elsewhere in the paper or supplements.

Resources

Persistent unique identifiers for research resources, RRIDs, can be used to track these key resources though the literature. They are requested or required in many journals, but even if your journal does not require them, we strongly encourage the use of these for the readers of papers who wish to easily track down the key materials like antibodies, software tools, or mice.

Software and Algorithms	*"The Kaplan-Meier estimation curve was visualized using Prism version 6.0.7 (GraphPad Software, MA, USA)."*	Prism suggested: PRISM (RRID:SCR_005375. Please make sure this RRID suggestion is accurate before including in your manuscript.<br>GraphPad suggested: GraphPad Prism, RRID:SCR_002798. Please make sure this RRID suggestion is accurate before including in your manuscript.

Generated by *SciScore*.
SciScore only screens the text of the methods section; we apologize if the tool has missed items reported elsewhere in the paper or supplements.

Transparency

?	Open code	If you wrote code to analyze your data, please consider sharing the code in a public repository. This makes it easier for others to reproduce your analyses, and may also aid others seeking to analyze similar datasets.
?	Open data	If permitted, sharing data on a public repository with a persistent identifier (a DOI or accession number) can improve reproducibility and make it easier for other scientists to expand on your work. Papers with open data are cited more often than papers without open data. Some institutions have an expert who can provide advice on data sharing.
		"The limitations of the presented study were as follows: first, our results are based on a

✓	Self-acknowledged limitations	*retrospective single-institutional study with a small sample size. Thus, patient characteristics might have influenced our data and we acknowledge limited generalizability. A prospective, multi-institutional study with a larger sample size is needed to validate the current results. Second, this study only included pathological stage IA LUAD. Thus, the positivity rate and clinical significance of the presence of MECA-79+ tumor cells in patients with pathological stages IBIV, with neoadjuvant or adjuvant therapy, and with non-adenocarcinoma non-small cell lung cancer should be investigated in further studies. Third, we did not have background data on gene alterations such as epidermal growth factor receptor, Kirsten rat sarcoma viral oncogene homolog, and anaplastic lymphoma kinase mutations. Thus, the relationship between tumoral MECA-79 expression and the genetic background will need to be clarifi..."*
?	Conflict of interest statement	i It is important to supply a conflict of interest statement, even if there are none to declare, to transparently present the research.
✓	Funding statement	i Thank you for reporting your funding source(s), or stating that there was no specific funding for this work.
n/a	Registration statement	i A registered protocol makes the planned research project more transparent and can protect against bias. Please consider registering future confirmatory studies.

Generated by ODDPub, limitation-recognizer, rtransparent, trial-identifier.

Figures

✓	No bar graphs of continuous data	i Thank you for not using bar graphs to present continuous data. Many different datasets and data distributions can lead to the same bar graph, and the actual data may suggest different conclusions from the summary statistics alone.
✓	No rainbow color maps	i Thank you for not using rainbow colormaps. Rainbow color maps distort readers' perception of the data by introducing visual artifacts and are not accessible to colorblind readers.

Generated by JetFighter, Barzooka.
