# Supplementary material for: Clinical and pathological implications of the presence of MECA-79-expressing tumor cells in pathological stage IA lung adenocarcinoma
Source: PLoS One. 2025 Oct 16;20(10):e0323233. doi: 10.1371/journal.pone.0323233 (PMC12530585; doi:10.1371/journal.pone.0323233)
Supplement: Attachment — Submitted filename: PLOS one review comment (MECA-79).docx [file pone.0323233.s003.docx]

PLOS one review comment

It was a great pleasure to review the manuscript “Clinical and pathological implications of the presence of MECA-79-expressing tumor cells in pathological stage ⅠA lung adenocarcinoma” by Tomohito Saito, et al. In this study, the authors investigated the relationship between MECA-79(+) tumors and prognosis in 195 patients who underwent lobectomy for stage 1A lung adenocarcinoma. They revealed that the solid size and invasive size of MECA-79(+) tumors were larger than MECA-79(-). Vascular invasion was also significantly frequent in MECA-79(+) tumors. In addition, Kaplan-Meier analysis showed that patients with MECA-79-positive lung adenocarcinoma had significantly worse recurrence-free survival.

However, this study includes several limitations, and its clinical significance appears limited. Therefore, the authors need to address several key issues to strengthen the manuscript.

Concerns:

#1. Regarding the patient selection process (Page 5, line 97-98. Page 15, line 273-276):

In this study, the inclusion criteria were a pathological diagnosis of primary invasive lung adenocarcinoma, pathological stage 1A. However, it is unclear why the authors selected only patients with stage 1A and adenocarcinoma. The clinical significance of limiting the study population to stage 1A lung adenocarcinoma should be clarified. If there are specific reasons for this selection, it should be stated explicitly in the manuscript.

#2. Regarding the statistical analysis (Page 7):

The descriptions of statistical analysis are insufficient. The authors should clearly specify which statistical tests were used to compare the two groups in the Methods section. In particular, it should be described which tests were applied for continuous variables and which were used for categorical variables.

#3. Regarding the small sample size.

In this study, the number of patients in the MECA-79(+) tumor group is only 10. Small sample sizes are prone to statistical instability and bias, which can lead to potentially unreliable results. Therefore, it may be worthwhile to consider including other histological types of lung cancer or more advanced stages to increase sample size and enhance the robustness of the findings.

Minor Points:

#1. (Page8: line184, Page10: Table 1)

The **presentation** of postoperative recurrence

→ The **localization** of postoperative recurrence
