# Supplementary material for: Clinical and pathological implications of the presence of MECA-79-expressing tumor cells in pathological stage IA lung adenocarcinoma
Source: PLoS One. 2025 Oct 16;20(10):e0323233. doi: 10.1371/journal.pone.0323233 (PMC12530585; doi:10.1371/journal.pone.0323233)
Supplement: Attachment — Submitted filename: Response_to_the_reviewers_ver_3.2.docx [file pone.0323233.s006.docx]

*AUTHORS’ RESPONSES TO COMMENTS FROM THE ACADEMIC EDITOR AND REVIEWERS*

Re: PONE-D-25-16991　“Clinical and pathological implications of the presence of MECA-79-expressing tumor cells in pathological stage IA lung adenocarcinoma”

Academic Editor:

Thank you for submitting your manuscript to PLOS ONE. After careful consideration, we feel that it has merit but does not fully meet PLOS ONE’s publication criteria as it currently stands. Therefore, we invite you to submit a revised version of the manuscript that addresses the points raised during the review process.

The reviewers provided constructive feedback, particularly regarding the statistical analysis, including the use of univariable and multivariable models given the limited number of events. Please incorporate more rigorous statistical methods to avoid potential overfitting and strengthen the validity of the findings. Furthermore, integrating a bioinformatic analysis of publicly available datasets to explore the expression of genes involved in MECA-79 glycan biosynthesis could provide additional mechanistic insights and further support the clinical relevance of MECA-79 in tumor biology.

*[Authors’ response]*

***Thank you for the constructive comments and valuable insights. We have revised our manuscript in response to the reviewers’ comments, as highlighted on the following pages. Moreover, we have revised the file names of figures, tables, and supporting information to comply with the journal’s requirements.***

***In response to the comments on our statistical methods from you and reviewer #3, we have excluded the multivariate logistic regression model including all candidate factors (Table 2 in the original version) from the manuscript to avoid overfitting.***

***While we acknowledge your concern regarding the limited number of events, our multivariate model with two selected variables represents a conservative approach that meets established statistical guidelines (i.e., the rule of ten events per variable). Although not optimal and providing limited support, this multivariate approach adjusts for confounding factors, which cannot be addressed using univariate analysis.*** ***Furthermore, performing Cox proportional hazards analysis while retaining the logistic regression models enables statistical triangulation, which potentially improves the reliability of the results.***

***Therefore, we have retained the multivariate logistic models including two variables in Table 3. In addition, we have conducted Cox proportional hazards regression analysis to evaluate the association of clinical and pathological characteristics with recurrence-free survival. We employed the same approach used in our logistic regression analysis by including only two candidate variables, including the presence of MECA-79^+^ tumor cells in the multivariate model, to follow the rule of ten events per variable (the number of recurrence or death events was 30).***

***We have revised the text in the Statistical analysis subsection of the Materials and Methods section as follows:***

***“Given the 17 postoperative recurrences observed within five postoperative years (as described below), two independent variables, including the presence of MECA-79^+^ tumor cells, were selected for multivariable model construction to avoid overfitting.”* [Page 9, Lines 195–198 of Marked Copy]**

***“Additionally, univariate and multivariate Cox proportional hazards regression models were used to evaluate the association of clinical and pathological characteristics with the RFS of patients with LUAD. We employed the same approach as that used in the logistic regression analysis, with only two candidate variables, including the presence of MECA-79^+^ tumor cells in the multivariate model, to follow the rule of ten events per variable (the number of recurrence or death events was 30, as described below) [28].”* [Page 9, Lines 201–207 of Marked Copy]**

***Additionally, we have revised Table 2 and added Tables 4 and 5.* [Pages 12-13, 16 and 18 of Marked Copy]**

***Furthermore, we analyzed the human lung adenocarcinoma dataset from The Cancer Genome Atlas database using cBio Cancer Genomics Portal and Survival Genie 2.0. This investigation focused on the mRNA expression of genes associated with MECA-79 biosynthesis and their association with overall survival. The majority of LUAD showed upregulation of genes associated with MECA-79 biosynthesis.***

***We have added the Analysis using publicly available genomic database subsection to the Materials and Methods section as below:***

***“Analysis using publicly available genomic databases***

***For a comprehensive examination of both gene expression patterns and survival outcomes, robust, publicly available cancer genomics resources were utilized. The LUAD dataset from The Cancer Genome Atlas (TCGA), which includes 565 cases and is a widely used resource for cancer genomics, was accessed via two platforms: the cBio Cancer Genomics Portal (https://www.cbioportal.org/) [20] and Survival Genie 2.0 (https://bhasinlab.bmi.emory.edu/SurvivalGenie2/home) [21].***

***The cBio Cancer Genomics Portal was used to analyze gene expression in LUAD tumor cells. Specifically, we examined the expression levels of genes associated with MECA-79 antigen biosynthesis (e.g., B3GNT3, B4GALT4, CHST2, and CHST4) [22–25]. Gene expression levels in LUAD tumor cells were compared with those of normal cells within TCGA LUAD dataset. In addition, co-expression analysis was performed to identify genes showing significant correlation (Spearman’s correlation coefficient > 0.5) with the expression of B3GNT3 and B4GALT4, providing insight into potential functional associations.***

***Survival Genie 2.0 platform was employed to analyze overall survival (OS) in TCGA LUAD cohort. Patients were stratified into high- and low-expression groups for each MECA-79–associated gene based on the median expression value.”* [Pages 7-8, Lines 160-177 of Marked Copy]**

***We have also revised the text in the Discussion section as follows:***

***“The MECA-79 biosynthetic pathway involves a series of glycosylation and sulfation steps that generate specialized O-glycan structures on proteins (Fig. 4A). Specifically, the enzymes beta-1,3-N-acetylglucosamine transferase 3 (B3GNT3) [22], carbohydrate sulfotransferase (CHST) family (CHST2 and CHST4)[23,24], and beta-1,4-galactosyltransferase 4 (B4GALT4)[25] have been shown to be essential for the production of MECA-79 antigen.***

***In an analysis of TCGA LUAD dataset (n = 565) using cBio Cancer Genomics Portal (https://www.cbioportal.org/) [Cerami E et al. Cancer Discov 2012], 87.5% of human LUAD samples showed gene alterations in B3GNT3, B4GALT4, CHST2, or CHST4, predominantly mRNA upregulation (78.6%), as shown for B3GNT3 and B4GALT4 in Fig. 4B. This, may contribute to tumoral expression of MECA-79 antigen in LUAD.”* [Pages 19-20, Lines 308-318 of Marked Copy]**

***“Further analysis of TCGA LUAD datasset using Survival Genie 2.0 (https://bhasinlab.bmi.emory.edu/SurvivalGenie2/home) [21] indicated that high expression (above the median) of B3GNT3 and B4GALT4 was associated with diminished OS (P = 0.008 and 0.005, respectively, as shown in Fig. 5). Furthermore, overexpression of B3GNT3 protein is associated with diminished disease-free survival and OS with advanced TNM stage [21,29,30], indicating the association of abnormal glycosylation with malignant transformation in LUAD.*” [Page 20, Lines 328-334 of Marked Copy]**

***“For example, analysis of TCGA LUAD dataset (n = 565) using the cBio Cancer Genomics Portal revealed that B3GNT3 is co-expressed with multiple genes associated with poor prognosis in patients with LUAD (Table 6) [33–42]. This finding suggests that MECA-79+ LUAD tumor cells may enhance their malignant potential”* [Page 21, Lines 358-362 of Marked Copy]**

***Additionally, we have added Figures 4 and 5 and Table 6.* [Pages 20, 20-21 and 22 of Marked Copy]**

Reviewer #1:

It was a great pleasure to review the manuscript “Clinical and pathological implications of the presence of MECA-79-expressing tumor cells in pathological stage ⅠA lung adenocarcinoma” by Tomohito Saito, et al. In this study, the authors investigated the relationship between MECA-79(+) tumors and prognosis in 195 patients who underwent lobectomy for stage 1A lung adenocarcinoma. They revealed that the solid size and invasive size of MECA-79(+) tumors were larger than MECA-79(-). Vascular invasion was also significantly frequent in MECA-79(+) tumors. In addition, Kaplan-Meier analysis showed that patients with MECA-79-positive lung adenocarcinoma had significantly worse recurrence-free survival.

However, this study includes several limitations, and its clinical significance appears limited. Therefore, the authors need to address several key issues to strengthen the manuscript.

Concerns: #1.

Regarding the patient selection process (Page 5, line 97-98. Page 15, line 273-276): In this study, the inclusion criteria were a pathological diagnosis of primary invasive lung adenocarcinoma, pathological stage 1A. However, it is unclear why the authors selected only patients with stage 1A and adenocarcinoma. The clinical significance of limiting the study population to stage 1A lung adenocarcinoma should be clarified. If there are specific reasons for this selection, it should be stated explicitly in the manuscript.

*[Authors’ response]*

***In the Introduction section of our manuscript, we highlighted the rate of postoperative recurrence for patients with pathological stage IA NSCLC is approximately 15%, which is equivalent to that of pathological stage II colorectal cancer. Our primary clinical question was why such a high proportion of patients with pathological stage IA NSCLC experience postoperative recurrence despite complete resection. The rationale for our inclusion criteria was as follows: We specifically analyzed pathological stage IA cases to minimize confounding variables associated with advanced disease stages. We also restricted our cohort to adenocarcinoma to eliminate potential confounding effects due to histological variations, given the known heterogeneity in biological behavior among NSCLC subtypes [Travis WD et al., J Thorac Oncol 2015]. Further, we included only patients who underwent complete (R0) resection with lobectomy to control for potential confounding effects related to surgical procedures or residual disease status.***

***In response to the reviewer’s comment, we have revised the Introduction section and Patient selection subsection in the Materials and Method section as below to further clarify the rationale for the inclusion criteria used in this study:***

***“Our primary clinical question is why such a high proportion of patients with pathological stage IA NSCLC experience postoperative recurrence despite complete resection. Delineation of the underlying mechanisms is essential for improving clinical outcomes.”* [Page 3, Lines 63–66 of Marked Copy]**

***“The rationale for our inclusion criteria was as follows: We specifically analyzed pathological stage IA cases to minimize confounding variables associated with advanced disease stages. Furthermore, we restricted our cohort to adenocarcinoma to eliminate potential confounding effects due to histological variations, given the known heterogeneity in biological behavior among NSCLC subtypes [13]. We only included the patients who underwent complete (R0) resection with lobectomy to control for confounding effects related to surgical procedures or residual disease status.”* [Page 5, Lines 105–111 of Marked Copy]**

***We have also cited an additional paper (Reference #13, Travis WD et al., J Thorac Oncol 2015) and updated the reference numbers accordingly.***

Reviewer #1:

Concerns: #2. Regarding the statistical analysis (Page 7):

The descriptions of statistical analysis are insufficient. The authors should clearly specify which statistical tests were used to compare the two groups in the Methods section. In particular, it should be described which tests were applied for continuous variables and which were used for categorical variables.

*[Authors’ response]*

***In response to the reviewer’s comment, we have revised the Statistical analysis subsection in the Materials and Methods section as below to further clarify which statistical test was used to compare the two groups:***

***“The Kruskal–Wallis test was used to analyze continuous variables, while Fisher’s exact test was used for categorical variables in comparing two groups.”* [Page 8, Lines 180–181 of Marked Copy]**

Reviewer #1:

Concerns #3. Regarding the small sample size.

In this study, the number of patients in the MECA-79(+) tumor group is only 10. Small sample sizes are prone to statistical instability and bias, which can lead to potentially unreliable results. Therefore, it may be worthwhile to consider including other histological types of lung cancer or more advanced stages to increase sample size and enhance the robustness of the findings.

*[Authors’ response]*

***Our aim was to investigate the risk of postoperative recurrence in patients with pStage IA LUAD. Thus, inclusion of other histological types or advanced stages are beyond the scope of this study. We agree, however, that other histological types or advanced stages of cancer should be investigated in future research.***

***In response to the reviewer’s comment, we have revised the Discussion section as follows:***

***“Thus, the clinical and pathological significance of the presence of MECA-79^+^ tumor cells in patients with more advanced pathological stages (i.e., IB–IV) or with other histological types, such as non-adenocarcinoma NSCLC (e.g., squamous cell carcinoma) should be investigated in future studies.”* [Page 23, Lines 372–376 of Marked Copy]**

Reviewer #1:

Minor Points: #1. (Page8: line184, Page10: Table 1)

The presentation of postoperative recurrence → The localization of postoperative recurrence

*[Authors’ response]*

***In response to the reviewer’s comment, we have revised description in Table 1 as follows: “Localization of postoperative recurrence”* [Page 11 of Marked Copy]**

***Additionally, we have revised the related description in the Results section as follows:***

***“The localization of postoperative recurrence was not significantly different between the two study groups (P = 0.77).”* [Page 10, Lines 221 of Marked Copy]**

Reviewer #2:

This is an automated report for PONE-D-25-16991. This report was solicited by the PLOS One editorial team and provided by ScreenIT.

ScreenIT is an independent group of scientists developing automated tools that analyze academic papers. A set of automated tools screened your submitted manuscript and provided the report below. Each tool was created by your academic colleagues with the goal of helping authors. The tools look for factors that are important for transparency, rigor and reproducibility, and we hope that the report might help you to improve reporting in your manuscript. Within the report you will find links to more information about the items that the tools check. These links include helpful papers, websites, or videos that explain why the item is important. While our screening tools aim to improve and maintain quality standards they may, on occasion, miss nuances specific to your study type or flag something incorrectly. Each tool has limitations that are described on the ScreenIT website. The tools screen the main file for the paper; they are not able to screen supplements stored in separate files. Please note that the Academic Editor had access to these comments while making a decision on your manuscript. The Academic Editor may ask that issues flagged in this report be addressed. If you would like to provide feedback on the ScreenIT tool, please email the team at ScreenIt@bih-charite.de. If you have questions or concerns about the review process, please contact the PLOS One office at plosone@plos.org.

*[Authors’ response]*

***We thank the editorial team for soliciting the report.***

Reviewer #3:

This retrospective study evaluated 195 patients with pathological stage IA lung adenocarcinoma and found that MECA-79 expression in tumor cells (10 cases (5.1%)) was associated with larger invasive tumor size, higher vascular invasion, and increased 5-year recurrence. Kaplan-Meier analysis showed significantly worse recurrence-free survival in MECA-79–positive cases, and multivariate analysis confirmed MECA-79 as an independent predictor of recurrence. These findings suggest that tumoral MECA-79 expression may contribute to a metastasis-promoting microenvironment and warrants validation in larger cohorts.

The authors acknowledged the small number of MECA-79(+) tumors as a key limitation of the study. In Tables 2 and 3, univariable and multivariable logistic regression analyses were used to evaluate clinical and pathological predictors of postoperative recurrence within 5 years. However, given the time-to-event nature of recurrence and survival outcomes, a Cox regression analysis with hazard ratios would be more appropriate than odds ratios.

Additionally, due to the limited number of recurrence or death events, it may be more appropriate to present univariable analysis only. The multivariable analysis shown in Table 3, which includes only two variables, provides limited support for the conclusions and may not be statistically robust.

*[Authors’ response]*

***We acknowledge the need of a statistically robust approach to validate our results, and this point is of paramount importance.***

***In response to the reviewer’s comment, we have excluded the multivariate logistic regression model including all candidate factors (Table 2 in the original version) from the manuscript to avoid overfitting.***

***While we acknowledge the reviewer's concern regarding the limited number of events, our multivariate model with two selected variables represents a conservative approach that meets established statistical guidelines (i.e., the rule of ten events per variable). Although not optimal and providing limited support, this multivariate approach adjusts for confounding factors, which cannot be achieved using univariate analysis. Therefore, we have retained the multivariate logistic models including two variables in Table 3.***

***In addition, we have conducted Cox proportional hazards regression analysis to evaluate the association of clinical and pathological characteristics with recurrence-free survival. We employed the same approach used in our logistic regression analysis by including only two candidate variables, including the presence of MECA-79^+^ tumor cells in the multivariate model, to follow the rule of ten events per variable (the number of recurrence or death events was 30).***

***We have revised the description in the Statistical analysis subsection of the Materials and Methods section as follows:***

***“******Given the 17 postoperative recurrences observed within five postoperative years (as described below), two independent variables, including the presence of MECA-79^+^ tumor cells, were selected for multivariable model construction to avoid overfitting.”* [Page 9, Lines 195–198 of Marked Copy]**

***“******Additionally, univariate and multivariate Cox proportional hazards regression models were used to evaluate the association of clinical and pathological characteristics with the RFS of patients with LUAD. We employed the same approach as that used in the logistic regression analysis, with only two candidate variables, including the presence of MECA-79^+^ tumor cells in the multivariate model, to follow the rule of ten events per variable (the number of recurrence or death events was 30, as described below) [28].”* [Page 9, Lines 201–207 of Marked Copy]**

***Additionally, we have revised Table 2 and added Tables 4 and 5.* [Pages 12-13, 16, and 18 of Marked Copy]**

***We have also revised the related descriptions in the Abstract, Results, and Discussion sections as follows:***

***“Using two variables per model, univariate and multivariate Cox regression models identified body mass index, International Association for the Study of Lung Cancer (IASLC) histological grade, vascular invasion, spread through air spaces, and presence of MECA-79+ tumor cells as prognostic candidates, with tumoral MECA-79+ status as an independent predictor except when combined with vascular invasion.”* [Pages 2-3, Lines 46–51 of Marked Copy]**

***“******Additionally, univariate Cox proportional hazards regression analysis identified body mass index (hazard ratio [HR] = 0.86, confidence interval [CI] 0.76–0.96, P = 0.009), IASLC grade of LUAD (HR = 2.23, 95% CI 1.05–4.73, P = 0.036), vascular invasion (HR = 3.31, 95% CI 1.51–7.25, P = 0.003), STAS (HR=4.34, 95%CI 2.11–8.91, P < 0.001), and presence of MECA-79^+^ tumor cells (HR = 4.38, 95% 1.51-12.7, P = 0.007) as candidate prognostic factors of RFS (Table 4)”* [Page 16, Lines 264–269 of Marked Copy]**

***“A prospective, multi-institutional study with a larger sample size, enabling rigorous multivariate statistical analysis, is needed to validate the current results.”* [Page 23, Lines 370-372 of Marked Copy]**

Reviewer #3:

For Figure 3, please include the number of patients at risk beneath the survival curves to enhance clarity and interpretability.

*[Authors’ response]*

***We have revised Figure 3 accordingly.***

Reviewer #3:

Lastly, there is a discrepancy between the study period and number of patients described in the Figure 1 legend, compared to what is reported in the figure itself and the main text. Please revise to ensure consistency across the manuscript.

*[Authors’ response]*

***We have corrected the legend of Figure 1 as follows:***

***“Briefly, out of 1014 patients undergoing surgery for lung cancer at Kansai Medical University Hospital between January 1, 2009, and January 31, 2017, a total of 195 patients who underwent complete resection with lobectomy for pathological stage IA invasive adenocarcinoma of the lung with no neoadjuvant therapy were included in this study.”* [Pages 5-6, Lines 118–123 of Marked Copy]**

***We have noticed the discrepancy between the version of the TNM classification system presented in the manuscript body and that in Table 1, the latter of which has been correct. We have also revised the Patient selection section in the Materials and Methods section as follows:***

***“Pathological stage IA (as per the 9th edition of the TNM classification system)”* [Page 5, Line 103 of Marked Copy]**

***We have also noticed that 6-sulfo sLe^X^ had been previously referred to simply as sLe^X^, we have revised the description to accurately specify 6-sulfo sLe^X^ as follows:***

***“However, the anti-metastatic role of tumoral 6-sulfo sLeX has also been shown in oral squamous cell carcinoma [32]. Tumoral 6-sulfo sLeX can attract CD8+ cytotoxic T lymphocytes, promoting the formation of immune synapses.”* [Page 21, Lines 352-355 of Marked Copy]**
