# Supplementary material for: Clinical and pathological implications of the presence of MECA-79-expressing tumor cells in pathological stage IA lung adenocarcinoma
Source: PLoS One. 2025 Oct 16;20(10):e0323233. doi: 10.1371/journal.pone.0323233 (PMC12530585; doi:10.1371/journal.pone.0323233)
Supplement: Attachment — Submitted filename: Response_to_the_reviewers_ver_4.2.docx [file pone.0323233.s007.docx]

*AUTHORS’ RESPONSES TO COMMENTS FROM THE ACADEMIC EDITOR AND REVIEWERS*

PONE-D-25-16991R1 “Clinical and pathological implications of the presence of MECA-79-expressing tumor cells inpathological stage IA lung adenocarcinoma”

Reviewer #3:

Saito et al. investigated the prognostic significance of ectopic MECA-79 expression inpathological stage IA lung adenocarcinoma. While the topic is clinically relevant, the revisedmanuscript still contains fundamental flaws that undermine its conclusions due to statistical fragility from severe sample imbalance, inconsistent analyses, and contradictory TCGA data.

Here are the concerns that need to be addressed:

1) The MECA-79^+^ group is too small (n=10), making the study susceptible to Type I errors or anecdotal results. The “rule of ten” cited by authors prevents convergence issues but does not guarantee against overfitting with such an imbalance (10 vs. 185 patients). This fragility is clearly demonstrated in their own Cox proportional hazards regression analysis. MECA-79’s prognostic significance disappears (p=0.067) when combined with vascular invasion, suggesting collinearity between MECA-79 expression and vascular invasion, rather than independent prediction. Furthermore, the author's author’s use of “statistical triangulation” through both logistic and Cox regression is unconvincing, as different model and variable combinations lead to inconsistent conclusions. This inconsistency reveals the instability of the findings rather than their reliability.

*[Authors’ response]*

*We appreciate the reviewer’s thoughtful critique. We agree that the MECA-79^+^ group is small and that this imbalance limits the robustness of our conclusions. We therefore present univariable Cox regression as the primary analysis in the main text and have moved the multivariable Cox models to the Supplementary Materials and have removed logistic models to avoid overstating fragile results. We further acknowledge the statistical fragility in the Limitations.*

*In response to the Reviewer’s comment, we have revised the Limitations of the study as follows:*

***“First, our results are based on a retrospective single-institutional study with a small sample size, particularly the MECA-79⁺ group (n = 10). This imbalance increases statistical fragility, limits power, and raises the possibility of collinearity with vascular invasion, which may confound the apparent prognostic effect of MECA-79. Therefore, the present study should be regarded as exploratory, and validation in a prospective, multi-institutional study with a larger sample size—ideally including stratification by vascular invasion—is required.”* [Page 16, Lines 310–317 of Marked Copy]**

2) The authors added a bioinformatic analysis using the TCGA database. However, this analysis critically weakens their central hypothesis. The authors report that 78.6% of LUAD samples in the TCGA showed mRNA upregulation of genes related to MECA-79 biosynthesis. In contrast, their own analysis found that only 5.1% of patients (10 of 195) showed actual MECA-79 epitope expression. This major discrepancy between high mRNA expression and low protein/epitope presence makes TCGA data inappropriate as supportive evidence.

*[Authors’ response]*

*We thank the reviewer for this important observation. We agree that TCGA analysis was initially added in response to the Associate Editor’s suggestion, does not provide supportive evidence for the prognostic value of MECA-79. On the contrary, the discrepancy between frequent upregulation of biosynthetic enzymes in TCGA (78.6%) and the rare detection of MECA-79 epitopes in our cohort (5.1%) highlights a limitation of our study and reflects a broader challenge in glycobiology. As the reviewer correctly noted, this discordance weakens the hypothesis that transcriptional upregulation alone can predict MECA-79 epitope expression, because carrier proteins are also required for the display of the glycan epitop. We have now clarified in the Discussion section that this discrepancy reflects a well-recognized phenomenon in glycobiology: mRNA levels of glycosylation enzymes do not necessarily correspond to the presence of glycan epitopes, due to post-transcriptional regulation and the requirement of carrier proteins (Neelamegham et al., Glycobiology 2016; Bennun et al., PLOS Comput Biol 2013). Accordingly, we explicitly state that TCGA findings should be regarded only as contextual mechanistic background rather than prognostic evidence.*

*In response to the reviewer’s comment, we have revised the Discussion sections as follows:*

***“While such upregulation may provide a biological background for MECA-79 biosynthesis, MECA-79 epitope expression was rare (5.1%) in our cohort, despite frequent mRNA upregulation in TCGA. This discrepancy highlights a well-recognized phenomenon in glycobiology: mRNA levels of glycosylation enzymes do not necessarily correspond to the presence of glycan epitopes due to post-transcriptional regulation and the requirement of carrier proteins [25,26]. These findings suggest that TCGA data should be regarded as contextual mechanistic background rather than prognostic evidence. Nevertheless, future studies incorporating glycoproteomic profiling are needed to resolve this gap.****”*　 **[Page 14, Lines 268–277 of Marked Copy]**

3) The authors limited their cohort to pStage IA LUAD to “minimizing confounding variables.” While this is a reasonable approach, they use survival data from the TCGA cohort, which includes relatively advanced stages, to support their claims about the prognostic value of MECA-79-related genes. This is methodologically inconsistent.

*[Authors’ response]*

*We agree with the reviewer that using TCGA survival data, which includes more advanced stages, was methodologically inconsistent with our focus on pStage IA LUAD. Accordingly, we have removed the description linking MECA-79–related enzyme mRNA expression with prognosis, and retained only the discussion focusing on the mechanistic background of MECA-79 expression. Accodingly, we have renamed Table 6 in the original version as Table 3 in the revised version.*

*In the revised Materials and Methods section, we clarify that the purpose of accessing the TCGA LUAD dataset was to explore the mechanistic background of tumoral MECA-79 expression, rather than its survival implications. The revised text reads as follows:*

***“For a comprehensive examination of gene expression patterns focusing on the mechanistic background of tumoral MECA-79 expression, a robust, publicly available cancer genomics resource was used. The LUAD dataset from The Cancer Genome Atlas (TCGA), which includes 565 cases and is a widely used resource for cancer genomics, was accessed via the cBio Cancer Genomics Portal (https://www.cbioportal.org/) [19].”* [Page 16, Lines 310–317 of Marked Copy]**

*Additionally, we have removed Figure 5 and all related descriptions concerning survival implications from TCGA dataset.*

*We have also revised the limitations of the study as follows:*

***“Fourth, TCGA analysis revealed frequent upregulation of biosynthetic enzymes despite rare MECA-79 epitope expression in our cohort. This discrepancy reflects a common disconnect between transcript levels and glycan presentation, underscoring that TCGA findings cannot be considered supportive prognostic evidence but only contextual background.”* [Page 17, Lines 325–329 of Marked Copy]**

4) The manuscript’s introduction compares the 15% recurrence rate of pStage IA NSCLC to that of pStage II colorectal cancer. This comparison is clinically irrelevant and should be removed. TNM staging systems are uniquely calibrated for each cancer type based on its specific anatomical and biological behavior. A direct comparison of survival rates between different stages of different cancers is meaningless.

*[Authors’ response]*

*We appreciate the reviewer’s comment and agree that comparing recurrence rates across different cancer types is clinically inappropriate. Accordingly, we have removed the cross-cancer comparison from the Introduction. In response to the reviewer’s comment, we deleted the sentence comparing the recurrence rate of pStage IA NSCLC with that of pStage II colorectal cancer.*

5) In their response to Reviewer #1, the authors state, “The Kruskal-Wallis test was used to… in comparing two groups.” The Kruskal-Wallis test is a non-parametric method for comparing three or more groups. For a two-group comparison, the Mann-Whitney U test is appropriate. This fundamental error in reporting the statistical methods further erodes confidence in the overall statistical rigor of the study.

*[Authors’ response]*

*We sincerely apologize for this error. We have corrected the text to report the* ***Mann–Whitney U test*** *for two-group comparisons. The numerical results remain unchanged.*

*In response to the reviewer’s comment, we have revised the Materials and Methods section as follows:*

***“******The Mann–Whitney U test was used to analyze continuous variables, while Fisher’s exact test was used for categorical variables in comparing two groups.”* [Page 8, Lines 169–170 of Marked Copy]**

6) Given that recurrence and survival represent time-to-event outcomes, logistic regression analysis is less appropriate than the Cox regression model. However, considering the severely limited sample size, the authors should focus on the time-to-event analysis with univariable analysis only to avoid overfitting and ensure statistical validity.

*[Authors’ response]*

*We thank the reviewer for this comment and agree that Cox regression is the appropriate method for time-to-event outcomes such as recurrence and survival. As noted above, we now present univariable Cox regression as the primary survival analysis. Multivariable Cox regression including is retained only in the Supplementary Material as an exploratory analysis to maintain transparency while avoiding overfitting in the main text, whereas logistic regression analyses have been removed.*

*We have revised the Materials and Methods section as follows:*

***“Univariable Cox proportional hazards regression was primarily used to evaluate the association of clinical and pathological characteristics with recurrence-free survival (RFS) in patients with LUAD. Significant factors (P < 0.05) in the univariable analysis were considered candidate risk factors. To minimize overfitting given the limited number of recurrence or death events (n = 3), multivariable Cox models –restricted to two candidate variables, including the presence of MECA-79^+^ tumor cells–were prespecified as exploratory analyses and are presented in the Supplementary Material (S1 Table).”* [Page 8, Lines 181–188 of Marked Copy]**

*Accordingly, Tables 2 and 3, which presented the logistic regression analyses, have been removed. Table 4 in the original version, which presented the univariable Cox regression analysis, has been retained and renumbered as Table 2 in the revised version. Table 5 in the original version has been moved to the Supplementary Material as S1 Table. In addition, we revised the Results section to clarify that the multivariable Cox models are exploratory analyses, as follows:*

***“Additionally, results of exploratory multivariable Cox proportional hazards regression analyses are provided in the Supplementary Material (S1 Table).****”* **[Page 12, Lines 240–241 of Marked Copy]**

*We have reported the results of exploratory multivariable Cox analyses in the Supplementary Material as follows:*
***“In exploratory analyses, the presence of MECA-79^+^ tumor cells showed a tendency toward association with worse RFS in some multivariable Cox proportional hazards regression models; however, this association was not consistent and disappeared in the model incorporating vascular invasion.”* [Page 2, Lines 6-8 of Supplementary Material]**
